# Acute Liver Ischemia Caused by Vasculitis Due to the Use of Synthetic Cannabinoids

**DOI:** 10.5334/jbsr.3556

**Published:** 2024-04-29

**Authors:** Ertugrul Cakir, Serdar Aslan, Esra Ibis

**Affiliations:** 1 Faculty of Medicine, Department of Radiology, Giresun University, Giresun, Turkey; 2 Faculty of Medicine, Department of Radiology, Giresun University, Giresun, Turkey; 3 Faculty of Medicine, Department of Radiology, Giresun University, Giresun, Turkey

**Keywords:** Liver ischemia, synthetic cannabinoids, vasculitis, computer tomography, emergency department

## Abstract

*Teaching Point*: Synthetic cannabinoids are drugs whose use has increased significantly in recent years and whose toxicological effects cannot be ignored. Chronic inflammatory processes such as vasculitis that may be caused by these substances pose serious health problems at all ages.

A 20-year-old male convict patient was admitted to the emergency department with complaints of sudden onset of right upper quadrant pain, fever, nausea, and vomiting. The history revealed that he had been inhaling methamphetamine, consuming bonzai and using various drugs since the age of 15, and it was reported that he had been involved in many cases of assault and violence. Computed tomography (CT) showed an increase in liver volume, heterogeneity in parenchymal density, and the presence of diffuse hypodense areas in a patchy pattern. In addition, increased thickness of the gallbladder wall, dilatation, and irregularities of the intrahepatic bile ducts were noted. Localized narrowing of the dilated bile ducts was observed. (A) The arrow points to a thrombus in the left intrahepatic branch of the portal vein. (B) A large thrombus image in the lumen of the superior mesenteric vein is shown. (C) Aneurysms in the cystic artery are indicated by the arrow. Increased wall thickness and irregularities in the vessels are indicated by the white circle. (D) Thickening and irregularities in the hepatic artery wall in the coronal plane are visualized in the white circle. (E–F) Red areas in the 3D Anteroposterior (AP) and Posteroanterior (PA) images of the liver demonstrate areas of damaged and affected liver (Figure 1) (Video).

**Figure 1:**
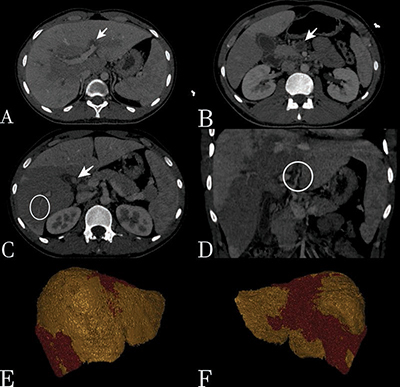
Acute Liver Ischemia and Vasculitic Findings.

The patient was diagnosed with acute liver ischemia due to vasculitis based on the elevated erythrocyte sedimentation rate and C-reactive protein levels, thickening and irregularities in the vessel walls, and venous thrombus findings, and treatment was started.

The patient responded well to nonsteroidal anti-inflammatory drugs and combined corticosteroid treatment.

The use of synthetic cannabinoids, which harbor major symptoms and serious side effects, has increased significantly in the young male population in the past years. The liver damage and vascular changes [1] as described in this patient, clearly demonstrate the potential harmful side effects of synthetic cannabinoids. Recently, studies investigating radiologic imaging of vasculitis have been published; however, the devastating effect of synthetic cannabinoid-induced vasculitis at a young age has not been previously demonstrated.
